# Optimizing beat synchronized running to music

**DOI:** 10.1371/journal.pone.0208702

**Published:** 2018-12-06

**Authors:** Jeska Buhmann, Bart Moens, Edith Van Dyck, Dobromir Dotov, Marc Leman

**Affiliations:** 1 IPEM, Ghent University, Ghent, Belgium; 2 LIVELab, McMaster University, Hamilton, Canada; University of California Merced, UNITED STATES

## Abstract

The use of music and specifically tempo-matched music has been shown to affect running performance. But can we maximize the synchronization of movements to music and does maximum synchronization influence kinematics and motivation? In this study, we explore the effect of different types of music-to-movement alignment strategies on phase coherence, cadence and motivation. These strategies were compared to a control condition where the music tempo was deliberately not aligned to the running cadence. Results show that without relative phase alignment, a negative mean asynchrony (NMA) of footfall timings with respect to the beats is obtained. This means that footfalls occurred slightly before the beat and that beats were anticipated. Convergence towards this NMA or preferred relative phase angle was facilitated when the first music beat of a new song started close to the step, which means that entrainment occurred. The results also show that using tempo and phase alignment, the relative phase can be manipulated or forced in a certain angle with a high degree of accuracy. Ensuring negative angles larger than NMA (step before beat) results in increased motivation and decreasing cadence. Running in NMA or preferred relative phase angles results in a null effect on cadence. Ensuring a positive phase angle with respect to NMA results in higher motivation and higher cadence. None of the manipulations resulted in change in perceived exhaustion or a change in velocity. Results also indicate that gender plays an important role when using forced phase algorithms: effects were more pronounced for the female population than for the male population. The implementation of the proposed alignment strategies and control of beat timing while running opens possibilities optimizing the individual running cadence and motivation.

## Introduction

Sports and exercise activities are generally believed to benefit from music listening. Under particular conditions music has been shown to capture attention, raise spirits, trigger a range of emotions, alter or regulate mood, evoke memories, increase work output, heighten arousal, induce states of higher functioning, reduce inhibitions, and encourage rhythmic movement [1, 2]. Effects of music during exercise can even be enhanced when certain types of music are considered [3, 4], especially when a certain level of synchrony between the musical stimuli and the listener's movements occurs [5–8]. A much-researched topic concerns the synchrony between music and running, measured by beat and footfall as markers of the rhythm that drives synchronized running. However, synchronizing one's footfall with the musical beat is not a straightforward endeavor. Some people easily synchronize, while others need an instruction to synchronize [9]. Some music facilitates movement (such as groove [10]) and running (such as activating music), while other music is less effective [3, 4]. In short, the use of music and specifically tempo-matched music has been shown to affect running performance, but it is an open question whether we can maximize the synchronization of movements to music and whether this maximum synchronization somehow influences kinematics and motivation.

In this context, we define synchronization as the stable maintenance over time of the sensorimotor coupling between beat and footfall. We define entrainment as the process that realizes the sensorimotor coupling, more specifically the process that brings the (perceived) beat and the (performed) footfall towards a stable timing. Rather than through pull and push forces (as in a dynamical system) it is straightforward to assume that prediction error minimization is a mechanism for entrainment [11]. For an in depth explanation of the factors that determine the strength of the sensorimotor coupling and entrainment, see Leman [12].

The tempo of running is expressed in steps per minute (SPM), i.e. cadence, and for music it is expressed in beats per minute (BPM). The tempo of running, or cadence, refers to the frequency of steps, whereas speed (expressed in km/h) refers to the total distance travelled in a certain amount of time. In previous studies, the preferred exercise intensity was often measured before the actual experiment and the music tempo was generally rather coarsely matched to a subject's spontaneous or comfort tempo (e.g., within a 10% range of the assessed cadence) [13, 14]. However, such an approach disregards the fact that the comfort tempo of an individual might be different at the time of the test, or that it might fluctuate during the test period. As a result, the contrast between the tempo of the music and the tempo of the exercise performance is likely to become too considerable to enable spontaneous entrainment. For that reason, we measure comfort tempo in the first part of each running task. In addition, a study by Van Dyck et al. [15] unveiled that uninstructed synchronization of running cadence to musical tempo occurs spontaneously when the tempo of the music does not deviate more than 2.5% from the initial running cadence. This finding highlights that conditions of entrainment yield affordances for sensorimotor adaptation to be effective, provided that the tempo of the music can be matched to the person's comfort tempo with high accuracy. Other research that addressed the particular relations between entrainment conditions and sensorimotor adaptation is, however, scarce [12, 16].

Another aspect that is likely to be important in terms of spontaneously manipulating running cadence is the anticipation effect reported in sensorimotor synchronization studies (SMS). For example, people typically tend to tap a little bit before the auditory stimulus, which is indicated by a slightly negative phase angle of the tap relative to the click, also referred to as negative mean asynchrony (NMA) [11]. They do this, presumably, because the sensing of the tapping has a longer delay than the sensing of the click [17]. Synchronization at brain level therefore means tapping before the click. Being able to continuously manipulate the exact moment of the beat might therefore prove to be a very accurate way to match and influence people's running capacity.

In the present study we focus on very precise sensorimotor (beat-footfall) alignment strategies during running. In fact, some of those strategies manipulate only the timing of the beat and it is expected that the timing of the footfall is somehow influenced by this manipulation. Moens et al. [16] describe four different strategies that manipulate sensorimotor alignment. However, these alignment strategies were not yet compared with each other within a single study, nor with the same musical test samples. Therefore, the purpose of this study is to contribute to a high definition sensorimotor alignment strategy that accounts for each person's sensorimotor ability. In particular, we aim at comparing different beat-footfall alignments using a randomized experimental setup where different strategies for supporting such alignments are applied. We hypothesized that strategies employing phase alignment to adjust the time of the musical beat relative to the time of the footfall during running would result in the highest level of sensorimotor synchronization and have a bigger effect on kinematics (cadence and speed) compared to strategies using period alignment instead. In addition, we expect that a high degree of synchronization would have an effect on motivation.

While our main aim is to discover whether different music-to-movement alignment strategies affect kinematics (cadence and speed) and/or motivation in distinct ways, we also want to address possible gender differences in entrainment capacity. The study by Van Dyck et al. [15] unveiled higher levels of entrainment for female compared to male runners. Other research shows that, when people are requested to rate the motivational qualities of musical excerpts, women pay closer attention to the rhythmical qualities of the stimuli compared to their male counterparts [18]. In addition, musical preference seems to affect women differently than men: when female runners listen to highly preferred stimuli, they tend to perform better than when listening to non-preferred music. In comparison, musical preference does not seem to affect the performance of male exercisers [19]. From the above-described findings, we expected to uncover differences between men and women regarding their ability to entrain and synchronize with the beats of the music. Such differences would be particularly relevant in terms of designing a more individualized music-technology approach.

## Materials and methods

### Subjects

To establish sample size, a power analysis for a repeated-measures design was conducted using G*Power 3.1.9.2 [20]. Based on a small effect size (.25) with alpha set at .05 and power at .95, it was estimated that at least 28 participants would be required. In total, 36 healthy, adult participants (19 males) took part in the study. All participants were recreational runners (M_age_ = 31.22 years; SD_age_ = 8.13 years) and indicated to be capable of running 30 minutes continuously. Of all participants, 38.89% were trained in music (Pearson Chi-Square test showed no significant relation between gender and musical background, χ^2^(1) = 1.22, p = .27). In addition, about half of them (55.56%) reported to generally run without music, 22.22% indicated to usually run with music, and 22.22% ran both with and without musical accompaniment. The study was approved by the Ethics Committee of the Faculty of Arts and Philosophy of Ghent University and was in accordance with the statements of the Declaration of Helsinki. Written informed consent was obtained from all the participants before the start of the experiment.

### Experimental design

#### Stimuli

A music database consisting of music tracks in the tempo range of 120–200 BPM (the range of natural running cadence) was created. The database included musical stimuli from a previous running experiment [15]. Using the Brunel Music Rating Inventory-2 (BMRI-2) test [21], all stimuli were rated as highly motivational for running. Additional tracks were selected to ensure complete coverage of the tempo range. In total, 43 tracks with clear beat information were selected. The tempo stability throughout each entire track was validated and intros lacking clear beats were cut from the stimuli using Audacity (http://audacity.sourceforge.net). BeatRoot [22] was applied to track the beats and tempo of each music track, while Adobe Audition (http://www.adobe.com) was used to normalize perceived loudness and minimize possible imbalances in sound pressure level using the ITU-R BS.1770-3 standard at -23 LUFS which is commonly used in audio broadcasts [23].

#### Apparatus

Data was collected using a 7" tablet (Panasonic FZ-M1) running Windows 8.1, which was strapped to a backpack. In addition, a pair of sensors, headphones, and a management computer was employed. The tablet operated as the main hub that handled incoming sensor data and provided the musical stimuli.

To detect footfall instants, participants were equipped with two iPods (4th generation); one attached at each ankle. Using the Sensor Monitor Pro application on the iPods, data from accelerometers and gyroscopes was streamed wirelessly to the tablet at a sampling rate of 100 Hz. Step timings were extracted from the signal using an approach based on Pappas et al. [24]. Speed measurements were performed using a sonar system (MaxBotix LV-MaxSonar-EZ: MB1010) connected to the tablet through a Teensy 3.1 micro-controller. It detected marker rods of 1.90 m high, placed at a regular interval of 10 m around the running track. Through computation of the time it took to cover each interval, absolute speed was determined. The analogue signal was sampled at 30 Hz and digitized using the Teensy.

The wireless connection between the tablet, iPods, and management computer was provided through a Wi-Fi router (TP-Link M5360), firmly strapped to the backpack, ensuring reliable communication between all crucial components. The management computer was applied to initiate the experimental sessions and to monitor sensor data in real-time. Musical tempi were manipulated using a phase vocoder based on the technology of Élastique Pitch of ZPlane.de [25]. The phase vocoder manipulates tempo in real-time without modifying pitch using a combination of frequency and time-domain methods. The system logged all data and calculations in real-time. Music tempo was adapted based on the selected alignment strategy (for the implementation of the music alignment strategies, see Moens et al. [16]). Finally, the aligned music was sent back to the participant using Sennheiser HD60 headphones connected to the tablet.

#### Procedure

All experiments took place in the Flanders Sports Arena of Ghent, Belgium. After being equipped, participants were asked to run on a 200 m running track for five minutes continuously, and this for six consecutive times. In each of the six 5-minute runs, a different alignment strategy was tested and it was ensured that all orders could occur only once.

Participants were instructed to run at their own comfortable pace. No information was distributed concerning the purpose of the experiment and all participants ran in solo conditions. After each 5-minute run, participants were allowed to take a break for several minutes in which they rated their perceived exertion (RPE) on the Borg Scale [26]. This way we could determine whether fatigue was influenced by the different music alignment strategies. In addition, they rated the level of physical enjoyment on the 8-item version of the Physical Activity Enjoyment Scale (PACES) [27, 28], a single factor scale to assess the level of enjoyment during a physical activity in adults across exercise modalities.

Each of the 5-minute runs started with 25 seconds of silence, followed by five musical excerpts of equal length (55 s) with an original tempo approaching the average cadence of the last seven footsteps. Musical tempo was then manipulated based on the selected alignment strategy, presented in the following section.

#### Conditions or alignment strategies

In the control alignment strategy (S0), music and running performance behave in a completely allochronic fashion (in our case: music is played 20 BPM faster/slower than the assessed running cadence). This strategy is used as a control condition to compare against five other music-to-movement alignment strategies.

Two strategies (S1, S2) involve the alignment of the music tempo to the runner’s cadence. The tempo matching occurs either at the beginning of a song only (S1), or continuously throughout the exercise (S2). Tempo-matching alone, however, does not consider the exact matching of the musical beats to the footfall instants; hence it neglects the phase (or the time between musical beat and footfall) while it maintains the period (for the musical beat and footfall, independently). In the adaptive tempo conditions, participants were still able to change their timing with respect to the beats as it takes several steps to determine the runners’ cadence.

Three strategies (S3, S4, S5) involve the alignment of the music tempo of the runner's cadence using relative phase angle manipulation. The relative phase angle expresses the footfall-beat time as a segment of the previous beat-period, which is defined as having 360°. Steps and beats are recurring events, hence cyclic in nature. Hence, the difference in timing between a beat and the nearest footfall can be expressed with a relative phase angle (-180° to +180°), where a 0° angle indicates that the beat and footfall instant coincide exactly. Alignment strategies that employ such phase manipulation minimize the relative phase angle between the beat and footfall instant, and therefore, they drive the alignment to any desired relative phase angle. In S3 the relative phase angle is minimized to 0° once, at the beginning of the exercise. The tempo of the music is however adapted continuously. The final two strategies (S4, S5) involve continuous relative phase angle adaptation starting at the beginning of the exercise. S4 guides the runner towards a predefined phase angle by adjusting the phase and hence tempo of the music at each step, thus at discrete timing intervals. In previous research, feedback from participants indicated that such a music adaptation sometimes felt unnatural or forced due to sudden tempo changes. Hence a new strategy is introduced (S5) based on adaptive oscillators [29], which results in continuous relative phase alignment towards a predefined relative phase angle using smoother tempo adaptations.

The first experiment in the present study intended that S4 and S5 guided the runner towards a 0° relative phase angle, or a perfect synchrony between footfalls and musical beats. However, after the experiment it became clear that an inaccuracy in the calibration led both alignment strategies towards -70° instead of 0°. This resulted in musical beats occurring after the footfall, while they were intended to occur simultaneously. For clarity, we henceforth refer to S4 and S5 as strategies with a configurable relative phase angle. A second (follow-up) experiment was then performed to assess the influence of our initially intended S4 [0°] and S5 [0°] strategies, which is reported subsequently. Based on the results of the first experiment, we also added an additional condition, which forced a +30° angle (beat before the step), and this is also reported later in this paper. In the discussion we consider the results from both experiments. A summary of the different strategies is provided in Table 1.

**Table 1:** Description of different alignment strategies.

Strategy	Short name	Type of music adaptation
S0	Allochronic music	Allochronic music (tempo differs at least 20 BPM from cadence), making it impossible to synchronize gait to music
S1	Fixed Tempo	One-time tempo matching at the beginning of a song
S2	Continuous tempo adaptation	Continuous tempo adaptation so BPM matches SPM each step
S3	Continuous tempo adaptation, in phase start.	Continuous tempo adaptation and in-sync (0° or -70°) relative phase start
S4 [x°]	Forced Relative Phase coherence–Algorithmic	Forced relative phase synchronization by continuous tempo alignment and relative phase adjustment towards configurable x° relative phase, updated each step. **Initial experiment conditions:** S4 [-70°] provided music where the beats occurred avg 60ms after the footfalls. **Follow-up experiment conditions:** S4 [0°] guided runners towards perfect phase synchrony with the beat.
S5 [x°]	Forced relative phase coherence–adaptive oscillator	Forced phase synchronization by continuous tempo and phase alignment towards configurable x° relative phase, updated using adaptive oscillators. **Initial experiment conditions:** S5 [-70°] provided music where the beats occurred avg 60ms after the footfalls. **Follow-up experiment conditions:** S5 [0°] guided runners towards perfect phase synchrony with the beat. S5 [+30°] provided music where the beats occurred avg 30ms prior to footfalls.

Although we are aware of the phenomenon of NMA in SMS studies, little is known about the magnitude of such a NMA for running and whether this is similar for all participants. We therefore decided to use 0° as a target relative phase and discuss our results taking into account the knowledge on NMA.

### Measurements

#### Cadence and speed

We examined the effect of the different alignment strategies on kinematic parameters such as cadence and speed. Average cadence and speed values during music playback are compared to those in the preceding 25 seconds of silence. The resulting dependent variables are expressed in percentages, where zero indicates no difference, while a negative or positive value indicates a respective decrease or increase in cadence or speed compared to the silent part of the condition.

#### Synchronization and phase angles

The level of synchronicity with the music, or rather, the stability and timing of the relation between a runner's footfall and the musical beat, is typically represented by the resultant vector or R. The length of this vector is a measure of tempo entrainment, ranging from zero to one with one representing perfect entrainment [30]. In addition, the angle of the resultant vector represents the average relative phase angle and reveals whether footfall instants occur before the beat is played (negative phase) or after (positive phase). The resultant vector represents an average of a time period, and is calculated based on a distribution of individual phase angles or timing differences between each step and the closest beat [31]. Each relative phase angle is calculated with the formula below, where *S*_*t*_ refers to a step at time *t*. *B*_1_ is the time of the beat that occurred before *S*_*t*_ and *B*_2_ is the time of the first beat after *S*_*t*_.

$$\varphi =360*\frac{S_{t}-B_{1}}{B_{2}-B_{1}}$$

Since 0° and 360° are in fact the same angles, the average relative phase angle is calculated with directional (or circular) statistics [30]. Our reference frame for calculating relative phase angles is the closest beat, so it makes sense to map the 0 to 360° relative phase to a -180° to 180° on the circular plane. Therefore, when φ was larger than 180° we transform it to the negative equivalent: $\varphi =\varphi -360\;if\left(\varphi >180\right)$. As such, a negative phase angle indicated the step occurred before the closest beat, while a positive phase angle indicates the step occurred after the closest beat. All data and plots are reported in this format.

### Data analysis

Each of the five music-to-movement alignment strategies was compared to the allochronic control strategy. Depending on how the data were distributed (normally or not), the analyses were either performed with repeated-measures ANOVA (e.g., for cadence and speed) or Friedman's ANOVA tests (e.g., for synchronization and motivation). When necessary, contrastive comparisons were performed (Wilcoxon signed-rank tests in case of non-parametric testing). Circular statistics equivalents where applicable (Wheeler-Watson Mardia tests are used for checking whether N circular distributions were homogeneous) [30, 32, 33].

## Results

### Synchronization

A Friedman's ANOVA showed a main effect of the strategy on resultant vector length, χ^2^(5) = 147.705, p < .001. Wilcoxon tests were used to follow up this finding. A Bonferroni correction was applied and so all effects are reported at a .003 level of significance. Results reveal that all six strategies differ with respect to the resultant vector length, except S2 versus S3 and S4 [-70°] versus S5 [-70°]. All comparisons are summarized in Table 2.

**Table 2:** Significant differences in phase coherence (resultant vector length R).

Comparisons (Mdn)	*z*	*p*^a^	*r*
S0 (0.03) vs. S1 (0.65)	-5.232	< .001	-.62
S0 (0.03) vs. S2 (0.80)	-5.232	< .001	-.62
S0 (0.03) vs. S3 (0.80)	-5.232	< .001	-.62
S0 (0.03) vs. S4 [-70°] (0.94)	-5.232	< .001	-.62
S0 (0.03) vs. S5 [-70°] (0.94)	-5.232	< .001	-.62
S1 (0.65) vs. S2 (0.80)	-3.268	.001	-.39
S1 (0.65) vs. S3 (0.80)	-3.991	< .001	-.47
S1 (0.65) vs. S4 [-70°] (0.94)	-5.232	< .001	-.62
S1 (0.65) vs. S5 [-70°] (0.94)	-5.232	< .001	-.62
S2 (0.80) vs. S3 (0.80)	-0.644	.519	-.08
S2 (0.80) vs. S4 [-70°] (0.94)	-5.185	< .001	-.61
S2 (0.80) vs. S5 [-70°] (0.94)	-5.059	< .001	-.60
S3 (0.80) vs. S4 [-70°] (0.94)	-5.122	< .001	-.60
S3 (0.80) vs. S5 [-70°] (0.94)	-5.001	< .001	-.59
S4 [-70°] (0.94) vs. S5 [-70°] (0.94)	-0.055	.956	-.01

^a^ *p* values were calculated with Wilcoxon signed-rank tests comparing all six alignment strategies with each other.

The circular Wheeler-Watson Mardia test was used to verify that all phase angle distributions were significantly different and thus not homogenous (W = 5113.6, p < .001), meaning the alignment strategies had a significant influence on the relative phase angles.

### Cadence

One of the dependent variables of interest is the change in cadence from running in silence to running with music. A 2x6 mixed-design ANOVA test with gender (male, female) as between-subjects variable and condition (S0 to S5 [-70°]) as within-subjects variable revealed a significant main effect of the strategy on the change in cadence, F(5,170) = 16.46, p < .001. Contrasts revealed that for S4 [-70°], F(1, 34) = 17.23, p < .001, r = .58, and S5 [-70°], F(1, 34) = 29.48, p < .001, r = .68, running cadence decreased significantly more (M = -1.81%, SE = 0.37, and M = -2.15%, SE = 0.34 respectively) compared to S0 (M = -0.53%, SE = 0.27).

There was no significant main effect of gender, indicating that on average there were no differences in change in cadence between male (M = -1.06%, SE = 0.15) and female participants (M = -0.93%, SE = 0.22), F(1,34) < 1, p = .78, r = .05.

However, an interaction effect between the strategy and the gender of the participant was observed, F(5, 170) = 4.97, p < .001, indicating that the change in cadence differed between men and women for different strategies. Contrasts were performed, revealing interaction effects between gender x S0 x S4 [-70°], F(1, 34) = 9.10, p = .005, r = .46, and gender x S0 x S5 [-70°], F(1, 34) = 6.40, p = .016, r = .40. This indicated that although, for both males and females, cadence decreased substantially during S4 [-70°] and S5 [-70°] compared to S0, this decrease was more pronounced for female runners.

### Speed

No main effect of the type of strategy on change in speed was uncovered, F(5, 165) = 1.02, p = .407, nor was there a significant main effect of gender, F(1, 33) = 1.36, p = .25, r = .20. Besides, there was no interaction effect between strategy and gender, F(5, 165) = 2.16, p = .061.

### Motivation and perceived exertion

No significant differences were revealed for perceived exertion (RPE) with a Friedman's ANOVA, χ^2^(5) = 6.64, p = .25.

Wilcoxon signed-rank tests (comparing each strategy with S0) were performed on the scores of the PACES scale. A Bonferroni correction was applied and so all effects are reported at a .01 level of significance. The motivational scores were higher for S5 [-70°] (Mdn = 71.38) compared to S0 (Mdn = 67.25), z = -2.662, p = .008, r = -.31. None of the other strategies displayed significant differences in motivation when compared to the allochronic strategy (S0). Table 3 summarizes the motivational comparisons. The median value for S5 [-70°] is not that different from S1-4. The reason, however, that ratings for S5 [-70°] are significantly different from S0, while the other alignment strategies are not, is due to differences in ranking: a higher percentage of the ratings where in favor of S5 [-70°] over S0 than in the other comparisons.

**Table 3:** Differences in motivation (PACES ratings).

Comparisons (Mdn)	*z*	*p*^a^	*r*
S0 (67.25) vs. S1 (69.88)	-.718	.487	-.08
S0 (67.25) vs. S2 (73.19)	-1.987	.048	-.23
S0 (67.25) vs. S3 (73.31)	-1.327	.196	-.15
S0 (67.25) vs. S4 [-70°] (71.75)	-1.581	.109	-.18
S0 (67.25) vs. S5 [-70°] (71.38)	-2.662	.008	-.31

^a^ *p* values were calculated with Wilcoxon signed-rank tests comparing five alignment strategies with the allochronic control condition (S0).

## Follow-up experiment

The data analysis of the first experiment showed surprising results of S4 and S5, leading to an investigation of the apparatus’ calibration. As we noted earlier, S4 and S5 were initially aimed to obtain a 0° phase synchronization. However, the system incorrectly forced a -70° relative phase angle. We believed that the non-intended forced -70° angle was the main cause of the observed cadence decrease and motivational increase for these strategies. Therefore, it was decided to do a follow-up experiment introducing correct 0° strategies. As the procedure and apparatus is almost identical, we only elaborate on the differences and results for cadence and motivation.

### Materials and methods

Tests took place at the same location of the first experiment. We recruited 11 of the initial participants (6 female, 5 male, M_age_ = 39.27 years; SD_age_ = 9.82 years) to have a similar population and to be able to compare both experiments.

Strategies S0, S1 and S2 were identical to the initial experiment. The -70° strategies (S3 [-70°], S4 [-70°], S5 [-70°]) were replaced by their intended counterparts (S3 [0°] S4 [0°] and S5 [0°]), where the beats coincide with the footfalls as initially intended. One additional strategy was added to explore possible inverse effects of the negative phase angle, namely S5 [+30°]. This strategy placed the beat slightly before the footfall, implying that the musical beats preceded the runner. For methodological reasons, this S5 [+30°] strategy occurred at the end of the experiment and was optional, to exclude influences of potential dropouts on our comparison with the earlier experiment. A summary of the different strategies is provided in Table 1.

### Results

All 11 participants completed all seven conditions. In total, two out of 77 trials were invalid due to sensor errors. Given our initial experimental results, this section focuses on phase angles, cadence, and motivation.

#### Phase angles and synchronization

As for the initial experiment, a repeated-measures ANOVA showed a main effect of the strategy on resultant vector length, F(6,54) = 103.794, p < .001. The circular Wheeler-Watson Mardia test was used to verify that all phase angle distributions were significantly different and thus not homogeneous (W = 1664.7, p < .001), meaning that the alignment strategies had a significant influence on the relative phase angles.

#### Cadence

In order to confirm that the retrieved effect on cadence from the initial experiment was due to the phase angle being targeted at -70°, we expected to find no effects on cadence for S4 and S5 targeted at 0°. Therefore, as in the initial experiment, a 2x6 mixed-design ANOVA test with gender (male, female) as between-subjects variable and condition (S0 to S5 [0°]; excluding S5 [+30°]) as within-subjects variable was performed. Indeed, no main effect of the strategy on the change in cadence was revealed, F(5,40) = 1.210, p = .322. In addition, no significant main effect of gender was observed, F(1,8) = 4.128, p = .077, r = .58, nor an interaction effect between the strategy and the gender of the participant, F(5, 40) = 1.149, p = .351.

When we included S5 [+30°], and performed a 2x7 mixed-design ANOVA, we did find a main effect on cadence, F(6,48) = 3.693, p = .004, and a small but significant gender effect, F(1,8) = 5.412, p = .048, r = .64. No gender x strategy interaction effect was found, F(6,48) = 1.306, p = .273.

Concerning the impact on cadence of different target phase angles in the configurable phase angle strategy (S5), additional tests were performed. To compare S5 [-70°] from our initial experiment with S5 [0°] from the follow-up experiment an independent samples t-test was executed. This test revealed that the decrease in cadence for S5 [-70°] was significantly larger (M = -2.15%, SE = 0.34) than for S5 [0°], (M = -0.09%, SE = 0.51), t(45) = -3.034, p = .004, r = .41. Furthermore, a paired samples t-test was used to compare S5 [+30°] with S5 [0°] from the follow-up experiment. Results showed that the change in cadence for S5 [+30°] was significantly larger and even positive (M = 0.68%, SE = 0.59) compared to S5 [0°], (M = -0.40%, SE = 0.44), t(9) = -2.970, p = .016, r = .70.

#### Motivation

Wilcoxon signed-rank tests (comparing each strategy with S0) were performed on the scores of the PACES scale. A Bonferroni correction was applied and so all effects are reported at a .008 level of significance. Only the motivational scores for S5 [+30°] showed a trend towards being significantly higher (Mdn = 44.00) than for S0 (Mdn = 38.00), z = -2.661, p = .008, r = -.57. No other differences were found. Table 4 summarizes the motivational comparisons.

**Table 4:** Differences in motivation (PACES ratings) for follow-up experiment.

Comparisons (Mdn)	*z*	*p*^a^	*r*
S0 (38.00) vs. S1 (42.00)	-1.721	.085	-.37
S0 (38.00) vs. S2 (42.00)	-1.736	.083	-.37
S0 (38.00) vs. S3 (41.00)	-0.949	.343	-.20
S0 (38.00) vs. S4 [0°] (41.00)	-1.961	.050	-.42
S0 (38.00) vs. S5 [0°] (42.00)	-1.661	.097	-.35
S0 (38.00) vs. S5 [+30°] (44.00)	-2.661	.008	-.57

^a^ *p* values were calculated with Wilcoxon signed-rank tests comparing six alignment strategies with the allochronic control condition (S0).

## Discussion

The goal of the present study is whether we maximize the synchronization of perceived musical beat and footfall during running, and whether this maximum synchronization influences kinematics and motivation. Table 5 summarizes the results, showing the different phase angle distributions for conditions and experiments. We then consider the effects of synchronization, cadence, and motivation. The last section discusses possible gender differences for these effects.

**Table 5:** Circular descriptives of the phase angle distributions for all strategies. Initial experiment data is abbreviated as I.E., while follow-up experiment data is abbreviated as F.U.E. For each alignment strategy the distribution of relative phase angles is described, using the following parameters: the mean angle *ø* representing the mean direction, the resultant vector length *R* and the circular variance *CV*, angular deviation *s* (dispersion around the mean), circular skewness *b* (asymmetry) and the circular kurtosis *k* (peak load). See [30] for more information.

Alignment Strategy	S0		S1		S2		S3		S4–70°	S4 0°	S5–70°	S5 0°	S5 30°
Short name	Allochronic Music		Fixed Tempo		Continuous Tempo Adaptation		Continuous Tempo Adaptation, in phase start		Forced Phase Coherence—Algorithmic		Forced Phase Coherence—Adaptive Oscillator		
Forced phase angle start / goal							-70°	0°	-70°	0°	-70°	0°	+30°
**Experiment**	I.E.	F.U.E.	I.E.	F.U.E.	I.E.	F.U.E.	I.E.	F.U.E.	I.E.	F.U.E.	I.E.	F.U.E.	F.U.E.
**N Steps**	26326	7246	26445	7285	26275	7451	26199	6709	25980	7466	25773	7419	6772
**N Songs**	180	55	180	55	180	55	180	50	180	55	180	55	50
**Mean relative phase angle *ø***	138°	165°	-13.7°	-17.7°	-8.04°	3.61°	-22.1°	-39.2°	-70.3°	-1.95°	-75.5°	-9.66°	17.4°
**Resultant Vector Length** |R|	0.0034	0.0085	0.424	0.402	0.196	0.136	0.671	0.667	0.911	0.912	0.925	0.936	0.895
**Circular Variance *CV***	0.997	0.991	0.576	0.598	0.804	0.864	0.329	0.333	0.0891	0.0877	0.0747	0.0645	0.105
**Angular Deviation *s***	1.4	1.4	1.1	1.1	1.3	1.3	0.81	0.82	0.42	0.42	0.39	0.36	0.46
**Circular Skewness *b***	0.00048	-0.0006	0.02	0.03	-0.014	-0.035	-0.031	0.024	-0.0049	0.0074	0.0011	0.02	0.011
**Circular Kurtosis *k***	-0.0016	0.0026	0.22	0.19	0.062	0.04	0.46	0.36	0.74	0.74	0.79	0.82	0.74

### Preferred phase angles, synchronization, and phase locking

**Fig 1:**
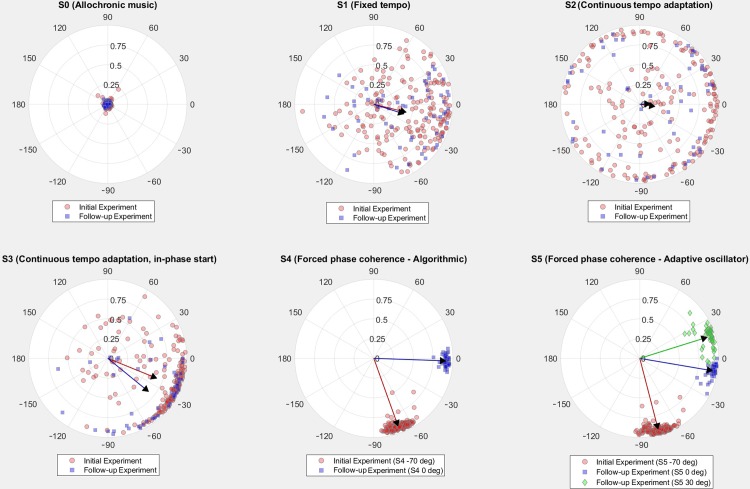
Circular scatter plots of resultant vectors. Each dot represents the resultant vector (both angle and length) per song per participant. A negative angle indicates that during that specific song, the footfall (averaged over the song) occurred before the musical beat (runner is first), while a positive angle indicates that the footfall occurred after the beat (music is first). The distance from the center indicates the resultant vector length. The closer the dot is to the circle perimeter, the higher the phase coherence, i.e. the more steps were taken towards this average phase angle. The arrows indicate the resultant vector length and average phase angle of the complete distribution of songs, thus providing a general overview of all participants’ behavior to all songs, in a specific condition. The color and form of each dot indicate whether the data point was from the initial or the follow-up experiment, which is denoted in the legend of the figures.

The data from the different alignment strategies reveal differences and preferences in synchronization behavior. Fig 1 provides a visual summary of the different strategies that were tested in terms of timing information of the step versus the beat (i.e. the relative phase angle) and the synchronization stability or phase coherence (i.e. resultant vector length).

In Fig 1, condition S0 clearly visualizes what the control condition was designed for, i.e. that runners don't synchronize, nor phase-lock (because they cannot). S0 can thus provide a baseline or comparison for later tests.

Since strategy S1, S2, and S3 did not use continuous relative phase angle manipulation, they gave a good idea of the (average over song) relative phase angle when runners ran with music at their (previously measured) preferred tempo. Once the song starts, the tempo remains constant (S1), or is continuously adapted to match the runners’ preferred tempo, which may change during the song (S2 and S3). These conditions allowed for *self-selected relative phase angles*. Since S2 and S3 used an adaptive tempo strategy, there was less freedom to deviate from the initial relative phase angle between footfall and beat (for S2 this relative phase angle is random, for S3 it is around -70° or 0° for initial and follow-up experiment).

Of particular interest was condition S1, where, due to the fixed tempo of the song, the highest degree of freedom was implemented with regard to the selection of the relative phase angle. Each song started at a random relative phase angle (random time with respect to footfall), but the distribution ended up with a clear clustering around negative relative phase angles (-14º and -18 º for initial and follow-up experiment respectively). In addition, there was a large spread in resultant vector lengths: in some cases people's footfalls showed a constant timing relative to the timing of the beats for a song (the dots on or near the circle perimeter, representing a high resultant vector length), in other cases this relation was less stable, or even almost absent (the dots close to the center of the circle, representing a low resultant vector length). This indicates that there was a convergence during the song from the starting angle towards the final angle. The average resulting relative phase angle, as indicated by the arrows in Fig 1 and the resultant vector length value (length of the arrow in Fig 1, see also Table 5), show a preferred relative phase angle around -15°. This confirms the preference for a slight negative mean asynchrony, and that runners prefer the footfalls occurring just prior to the beats.

In S2, the tempo of the music was continuously matched to the tempo of the runner. The figure shows high amounts of synchronization (runners consistently keeping their initial relative phase) but with a slight clustering around the same negative relative phase angles. This indicates that phase attraction towards the preferred relative phase angle found in S1 still happened with adaptive tempo conditions, but it is less pronounced and it occurs only when starting close to this preferred angle.

S3 further elaborated on this finding. The blue dots on Fig 1.S3 indicate data from the follow-up experiment, where each song started around 0°, that is, the beat is perfectly in sync with the footfall. These participants deviated towards -40°, thus increasing their relative phase angle. For the initial experiment, we discovered a reverse effect: participants started in -70° and finished in on average -22°, thus reducing their relative phase angle. This indicates that there is a ‘phase attractor’ between -20° and -40°, and that when the starting relative phase was close to these angles, participants tended to get phase-locked around their preferred relative phase angle.

Note that this attractor effect was not clearly visible in S2 as there was no clear clustering around these relative phase angles. In this condition, the starting relative phase angle was random. To explain why runners starting in these random relative phase angles were not attracted to their own preferred angle, we refer to a mathematical model which is used in movement coordination: the HKB model [34]. This model shows an attraction basin around the preferred relative phase angle, but if a relative phase angle deviates too far from this preferred relative phase angle, the attraction is not present. This could explain why there are high synchronization scores, or resultant vector lengths, at all different relative phase angles. Additionally, when participants were eventually attracted towards this preferred angle, another issue arose: since the songs lasted only 55 seconds, it is likely that runners did not have enough time to converge towards their preferred relative phase angle. Fig 1.S2, visualizes this phenomenon with lower resultant vector lengths between -90º and 0° (compared to S1), which can be attributed to this time-consuming convergence. A transition from one relative phase angle to a completely different relative phase angle results in a wide distribution and thus a lower resultant vector length. We assume that if the song would have been longer, the same clustering as in S1 and S3 might have been discovered in S2 for starting relative phase angles close to the preferred relative phase angles.

S1 and S3, both the initial and the follow-up experiments, clearly revealed a negative mean asynchrony (NMA), i.e. the participants showed a tendency to anticipate the beats (from -40° to -14°). This is in accordance with other studies concerning sensorimotor synchronization. On average, the runners put their feet down 40 to 15 ms before they perceive the musical beat. This asynchrony could be explained by the fact that the tactile signal takes longer than the audio signal to reach the brain. To synchronize in the brain, the tactile signal needs to occur before the audio signal, hence the asynchrony of about -15º in relative phase. This is in line with the nerve-conduction hypothesis, where peripheral processing time is dependent on the distance of feedback (tactile or auditory) to the brain [17] and the sensory accumulator model assuming that synchrony is established at the level of central representations [35]. Another group of explanations is based on the onset computation or P-center hypothesis [36]: if an event (e.g., a tap or a footstep) is extended over time, the perceptual center (P-center) differs from the onset of the event. For tapping, it is suggested that rather than the initial surface contact, the moment of peak force is the meaningful target in timing control [37].

In terms of attraction towards the NMA or preferred phase angle, we found that our results from S1—S3 are in line with Haken-Kelso-Bunz (HKB) model [34]. This model depicts interlimb coordination and phase transitions between different states. It shows a strong attraction basin around the 0° phase angle and a secondary attractor near 180° (antiphase), inducing phase transitions from the current angle towards the closest attractor. The model also shows that the strength of this attraction force decreases with increasing movement frequency, and at higher tempi (such as running at a higher cadence than 150 SPM) the secondary antiphase attractor disappears and only relative phases close to 0° get attracted. This is remarkably similar to the findings of S1 through S3 (although with a rotation towards the NMA or preferred relative phase angle of the participants): (1) if the starting phase differs enough from the NMA, no attraction is present when both systems operate at the same frequency (music is matched to cadence) and (2) when the starting phase is close to the NMA, participants converge towards their NMA.

S4 and S5 were designed to keep a constant relation between the moments of the footfall and the beat. This is clearly illustrated in Fig 1 by the concentrations of dots on the circle perimeter near the target phase angle that was implemented in these strategies: -70° in the initial experiment (in red), 0° (in blue) and +30° (in green) in the follow-up experiment. This indicates that the software works as configured, and that we can force users towards a certain relative phase angle. Later sections of the discussion will look into possible kinematic and motivational effects of certain relative phase angles.

To summarize, we found that the three conditions allowing *self-selected relative phase angles* show that the preferred angle does not approximate 0°, but is slightly negative–following the NMA displayed in sensorimotor synchronization experiments [11, 38]. We also found indications for an attraction force towards this preferred relative phase angle, especially when the initial relative phase was close to the preferred relative phase. When the initial starting relative phase was not near to this attractor, a stable relative phase was kept at this angle. Hence, to reach the preferred self-selected relative phase angle, the starting relative phase is important. If we want to design future music-to-movement alignment strategies that support the runners' natural and individual interactive behavior with music, not only should the technique be capable of selecting music that matches the preferred tempo of the runner, it should also be able to apply the preferred relative phase angle (as measured for instance with a fixed tempo strategy like S1 and fine-tuned with a strategy similar to S3) as a target relative phase angle in strategies similar to S4 and S5.

### Effect on cadence

**Fig 2:**
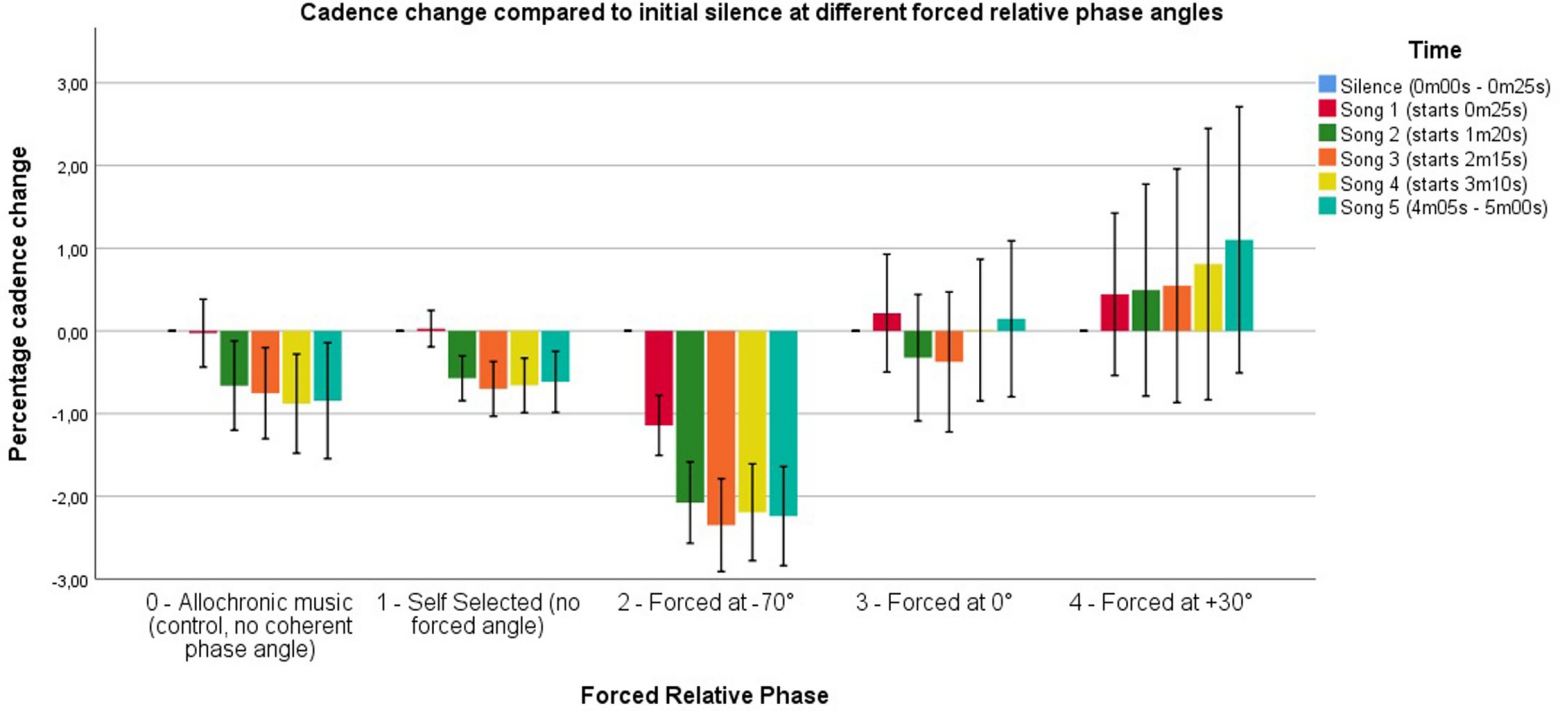
Cadence change compared to initial silence over time. Conditions are grouped based on the forced relative phase angle to improve visual representation. Self-selected relative phase angles include S1 through S3, Forced at -70° are S4 [-70°] and S5 [-70°], forced at 0° include S4 [0°] and S5 [0°], and finally forced at +30° is S5 [+30°]. Self-selected relative phase angles induce little to no cadence change compared to the control condition, while the -70°, 0° and +30° conditions have an effect over time. Error bars represent 1 SE. The error bars are noticeably higher in the 0° and +30° conditions, as the amount of participants (N = 11) for the follow-up experiment was lower than the amount of participants in the initial experiment (N = 39).

The combined results of the initial and the follow-up experiment indicate that strategy S1, S2, and S3 have no significant effect on cadence when compared to the allochronic music strategy (S0). Runners can be phase-locked, i.e. maintain a stable footfall-to-beat relation (seen best in S1, S2 and S3), and in general running with these strategies reflects a natural preference for a slightly negative phase angle relation (NMA). In contrast, our main results do show an effect of the alignment strategy on cadence with S4 and S5. This is further elaborated on with the follow-up experiment: the effect appears to be dependent on the target phase angle settings in the strategy. Fig 2 shows the resulting cadence change based on the forced relative phase angle.

For this discussion, we group several conditions based on the forced relative phase angle: (0) control or asynchronous music, (1) self-selected relative phase angles, (2) forced at -70°, (3) forced at 0°, and (4) forced at +30°. Fig 2 visualizes the effect of forced relative phase angles on cadence.

There was no difference between self-selected angles and the control condition in terms of the cadence change, meaning participants did not show any difference in running behavior in their self-selected angle compared to allochronic music. There was however an effect of 0°: cadence seemed to increase to a minor extent, while this increase was more pronounced at +30°. At -70°, we noticed a clear decrease in cadence. Thus, to have a neutral null-effect, the optimal relative phase angle would be the preferred relative phase angle as found in S1 and S3, similar to our earlier findings on synchronization.

The decrease in cadence observed in S4 and S5 at -70º could be explained by the fact that the beats were continuously perceived approximately 70 ms after the moment of the footfall. The participant was therefore constantly urged to lengthen the duration of his or her next step to reach the preferred -20° attractor. We theorize that, in order to deal with the perceived sensorimotor error, the brain continuously adapts in order to try to minimize the perceived sensorimotor error (i.e., obtain NMA). Although that error persists (due to our matching algorithm), the entrainment (or brain-driven sensorimotor error minimization) persists as well, and the outcome is a decrease in cadence. Conversely, the introduction of the beat prior to the footfall instant (positive relative phase angle) might rather induce a feeling of 'being late', which could in turn stimulate the runner to speed up. This was indeed observed in the follow-up experiment, where S5 at +30° resulted in an increase in cadence compared to S0.

To further explore these results, a correlation analysis was performed to see a potential relation between the relative phase angle and the cadence change. This analysis only considers the data of conditions where phase manipulation was used (S4 [-70°], S4 [0°], S5 [-70°], S5 [0°] and S5 [+30°]).

A Kolmogorov-Smirnov test indicated that the data were not normally distributed (p < .01), hence a Spearman's Rho correlation test was performed. We assumed that participants would have tried to reach their preferred relative phase angle around -20°, hence a one-tailed test was performed as this assumption implies that a negative relative phase angle would result in a lower cadence and vice versa. We uncovered a positive correlation between the relative phase angle and cadence change (r = .293, p < .001, n = 681), showing that a connection between cadence change and the forced relative phase angle indeed exists.

Furthermore, the influence of the relative phase angle appeared to build up over time. Fig 2 shows the evolution of cadence change for different strategies over the five songs. When looking at the average cadence change over time, it was evident that the influence was not immediate, but increased over time. The lowering-cadence effect seemed to stop around 2.2% slowdown (similar to the tempo basin found by Van Dyck et al. [15]), while the cadence increasing effect was less pronounced. This shows that the relative phase angle manipulation effect was 'limited' towards the influence on cadence.

### Motivation

In the initial experiment, as well as in the follow-up experiment, similar results were obtained when comparing the enjoyment ratings of the different strategies to S0. The strategies that did not significantly affect running cadence (S1, S2, S3, S4 [0°], and S5 [0°]) were not rated differently from S0. However, in the two conditions that affected cadence most (S5 [-70°] and S5 [+30°]) a small but significantly higher enjoyment rate, or a tendency towards such an effect, was observed. According to literature on gaming [39] and exergames [40] the balance between skill and challenge is crucial for being intrinsically self-rewarding. When challenged at a level that the gamer perceives as pleasant, he or she is more likely to have a positive experience. As such, it could be suggested that a small deviation from a preferred cadence, such as realized by continuously manipulating the relative phase angle to a non-preferred phase angle (S5–70° and +30°), might imply a more challenging or rewarding running exercise. A challenge that is not too demanding for the runner’s skills (note that no significant increases in RPE were observed) could result in a more pleasant and rewarding experience than when the runner is not challenged or manipulated to change his or her preferred running behavior. According to Fritz et al. [41] moving in synchrony with music can evoke a sense of agency, or a feeling of 'being in control'. Combined with a certain amount of physical exertion, this sense of agency might contribute to a perceived positivity bias [42]. As such, our results seem to suggest that finding a new phase angle balance induces such feelings of agency. Similar to the skill-challenge balance [39, 40], being in and out of balance with a slightly demanding phase angle seems to more positively impact enjoyment levels compared to maintaining a preferred, less demanding, phase angle. Alternatively, error minimization towards NMA can be seen as a regulatory mechanism to homeostasis (an equilibrium state consisting of predictive, expressive and effort processes) [12].

**Fig 3:**
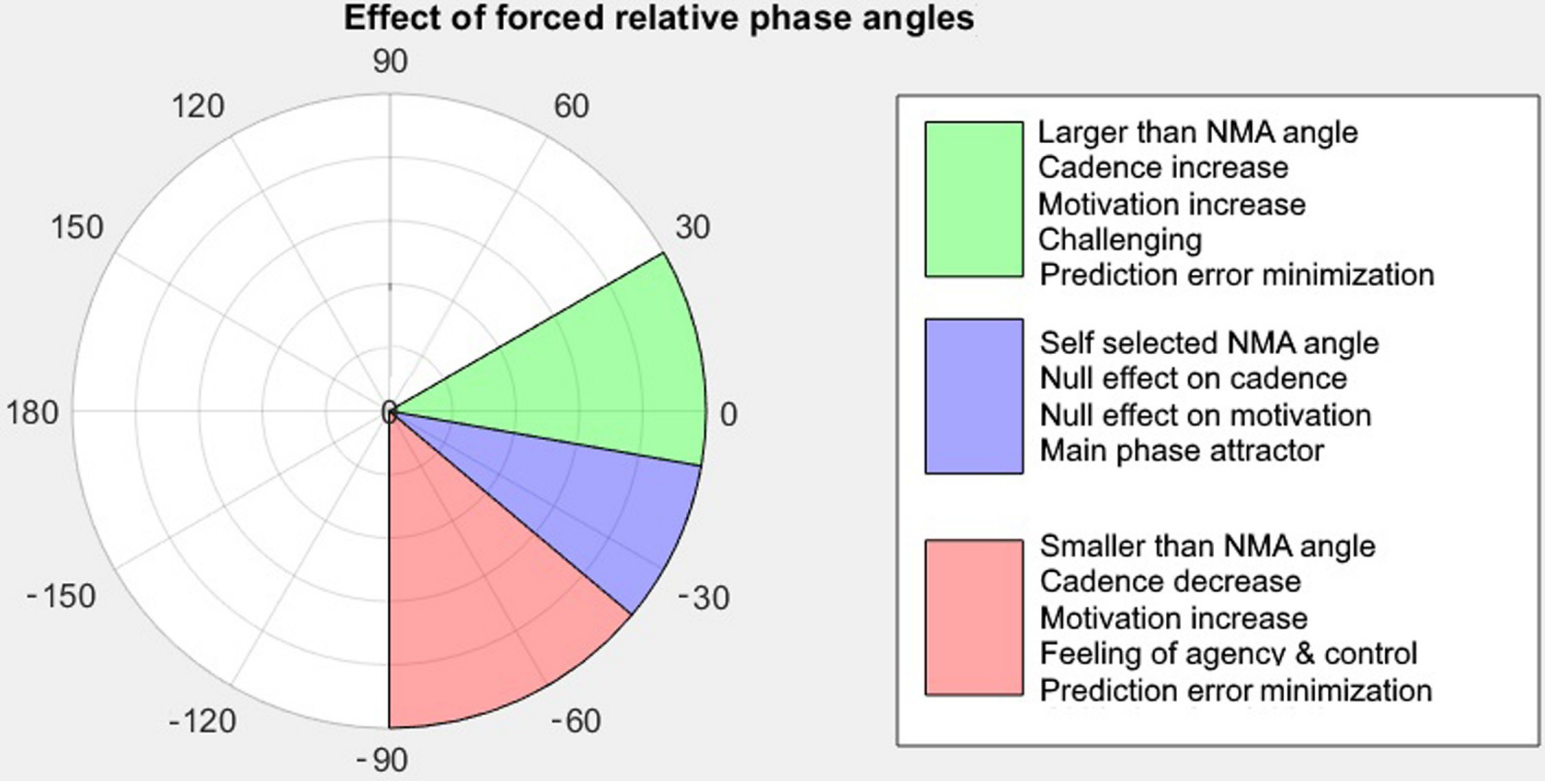
Summary of the effects of running in a forced relative phase angle.

Fig 3 summarizes our findings concerning the influence of forced relative phase angles. We note 3 regions: the neutral 'self-selected' or negative mean asynchrony near -20° (footfalls slightly before beats), the cadence increasing larger angles between -10° and 30° (steps simultaneous or after the beats) and the lower angles between -40° and -90° (steps noticeably before the beats). The effects of running in these regions are shown in the legend.

### Gender differences

Several studies gave direct and indirect proof or reason to expect differences in how men run, entrain, and synchronize with beats of the music, compared to women [15, 18, 19]. And although no main effect of gender was found on the change in cadence, some interesting interaction effects were uncovered that are worth discussing. Below, we compare the running behavior of men and women from the perspective of preferred phase angle and synchronization, cadence, and motivation.

#### Gender differences: Preferred phase angle and synchronization

**Fig 4:**
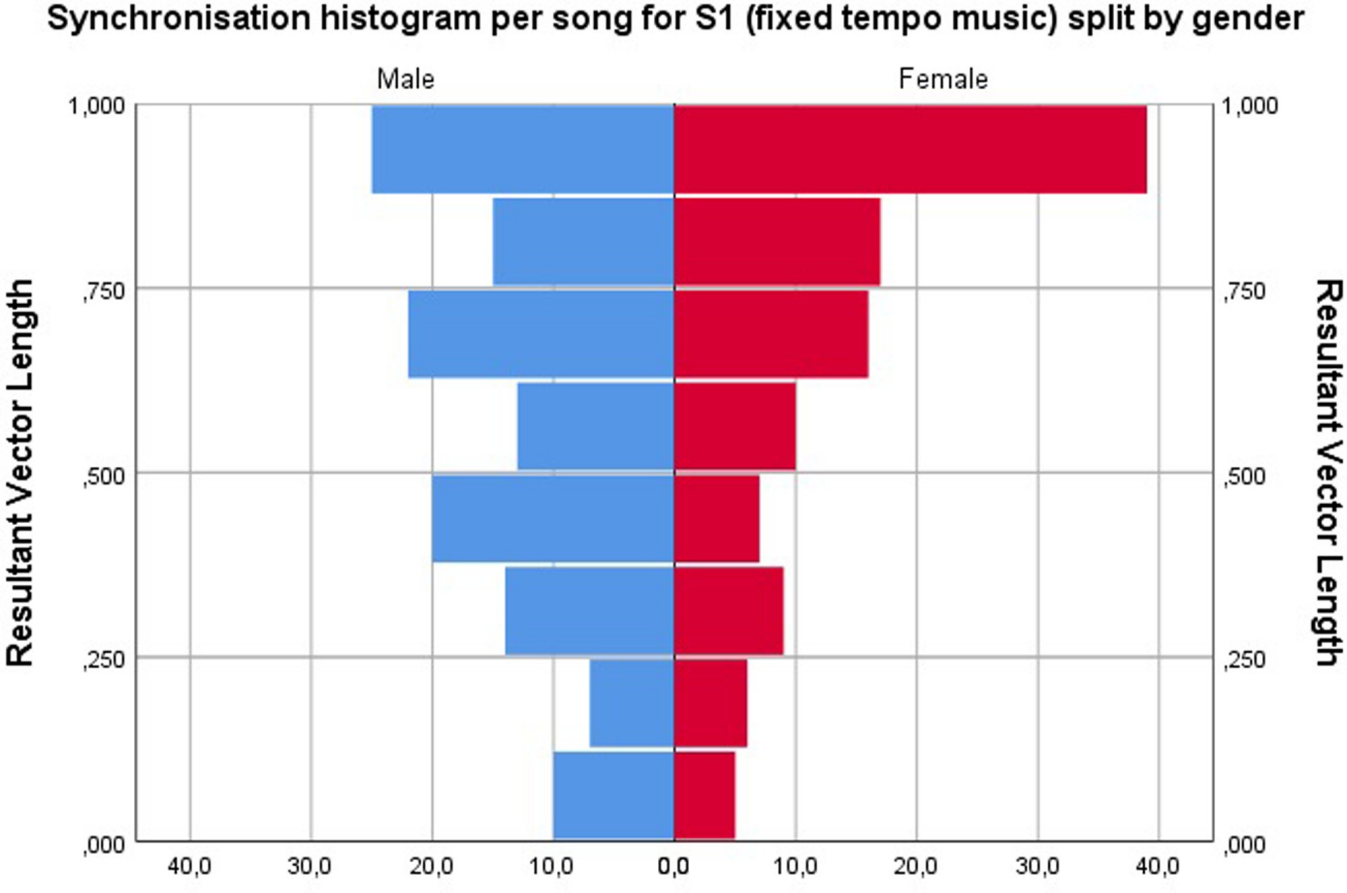
Resultant vector length or phase coherence for all songs in the S1 (fixed music) condition. A high level of phase coherence is observed for most of the women, whereas the flatter distribution of the resultant vector length for men indicates that a higher percentage of men did not maintain a phase-locked running behavior over the duration of a song.

Analysis showed that there is no significant difference between genders for average phase angles in all different conditions. However, in the fixed music condition (S1), where participants had the most freedom to self-select relative phase angles (and converge to their NMA), we noticed a slight difference in distributions for males versus females. The relative phase angle distribution was slightly more asymmetrical and wider than that for females, indicating a larger spread in relative phase angles and a less consistent synchronization. When looking at the phase coherence or synchronization of these angles (expressed by the resultant vector length), more pronounced differences emerge: women tend to reach higher synchronization scores than men in the fixed music (S1) condition. Fig 4 shows the distribution of the resultant vector lengths for S1.

Analysis with independent t-tests confirmed that there was indeed a significant difference in the resultant vector length for S1 (fixed tempo) for males (M = 0.57, SD = 0.27) compared to females (M = 0.68, SD = 0.27), t(233) = -2.964, p = .003. Females thus obtained higher synchronization scores. No other conditions showed such differences; perhaps because these conditions (S2-S5) allowed less freedom to self-select phase angles.

Using a resultant vector length of .75 as a threshold to determine running in phase coherence or not (similar to [4]), we see 52% of songs in phase coherence for women versus 23% for men in the S1 (fixed tempo) condition.

#### Gender differences: Cadence

A study on running to music [15] reported differences between male and female participants: women showed significantly higher levels of tempo entrainment than men. This is in line with research by Karageorghis [18], who found that women pay more attention to the rhythmic characteristics of music than men do. These findings make it interesting to look at the before mentioned forced-phase influence on cadence with the gender as a grouping factor.

**Fig 5:**
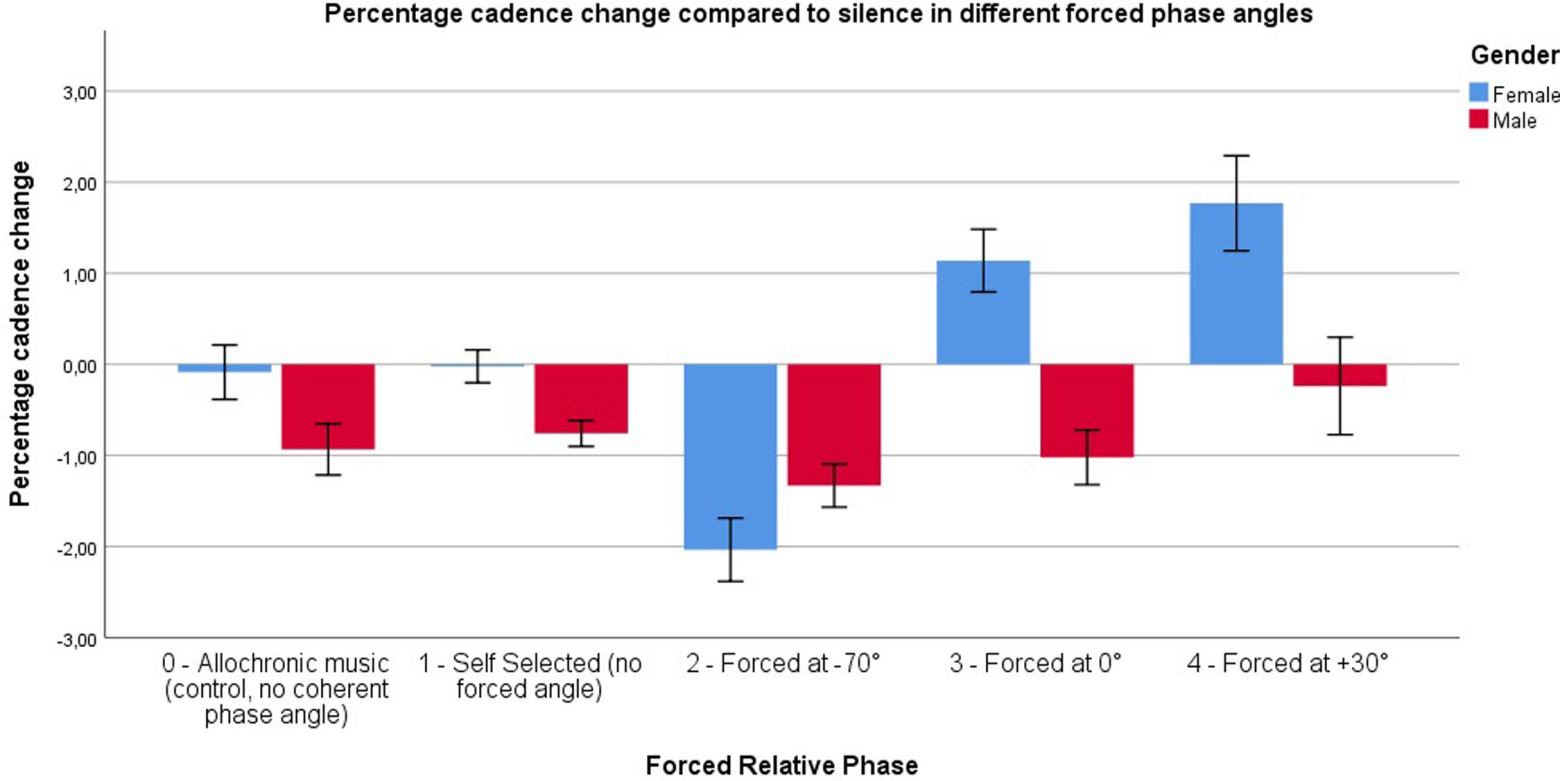
Cadence change compared to initial silence, split on gender. Conditions are grouped based on the forced relative phase angle to improve visual representation. *Self-selected angles* include S1 through S3, *Forced at -70°* are S4 [-70°] and S5 [-70°], *Forced at 0°* include S4 [0°] and S5 [0°], and finally *Forced at +30°* is S5 [+30°]. Both the control and self-selected phase angles induced little to no cadence change, while the -70° and +30° conditions had an effect that was more pronounced for females. Running at 0° phase angle seemed to have neutral effects for the males but a cadence increasing effect for females.

The main results showed an interaction effect between condition (alignment strategy) and gender (F(5, 170) = 4.97, p < .001) for the cadence change, indicating that the change in cadence differed between men and women for the different strategies. More in depth analysis showed a more pronounced change in cadence between the strategies for female runners (see Fig 5). It seems that, compared to their male counterparts, women demonstrated lower levels in cadence change with S4 [-70°] and S5 [-70°], and higher ones when running with S1, S2, and S3.

**Fig 6:**
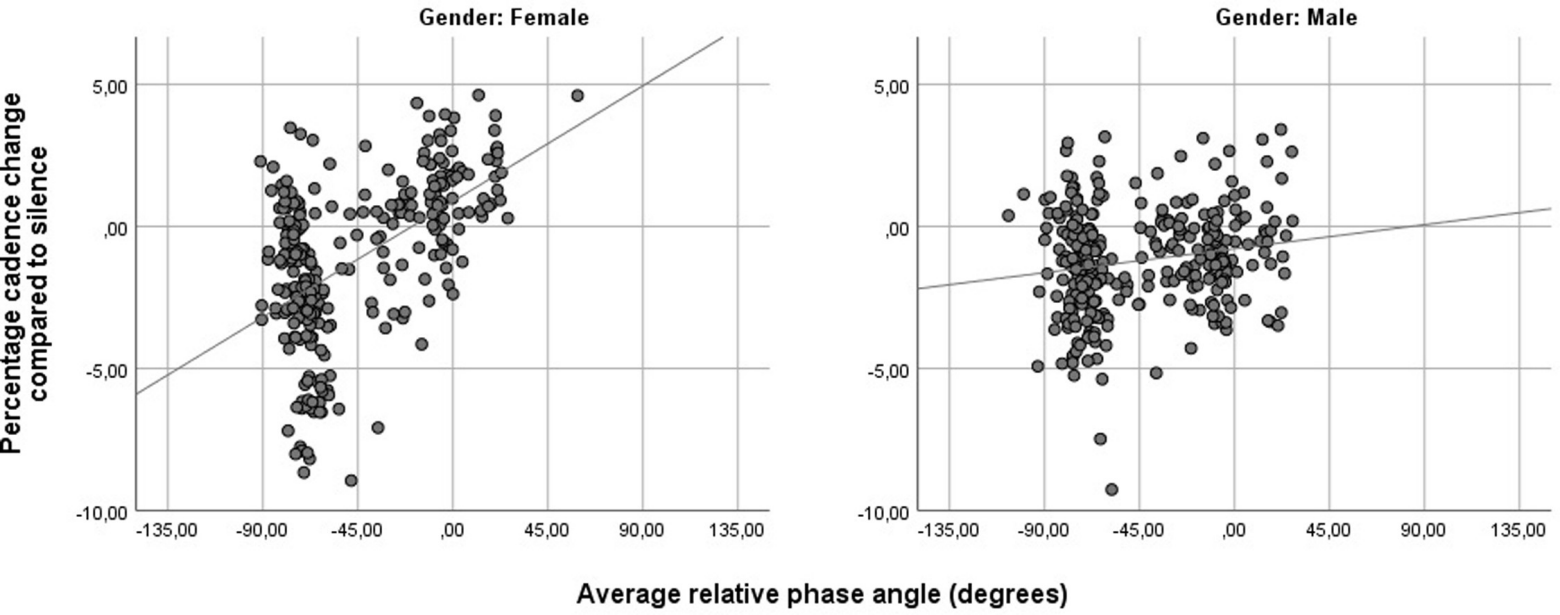
Correlations between forced relative phase angle and cadence change. The effect is more visible for the female than the male population.

A correlation test was performed on the forced relative phase angle conditions to test the significance of the forced phase angle on cadence change. Fig 6 illustrates the dataset. Both male and female distributions were non-normal (p < .01) as determined by the Kolmogorov-Smirnov test. The Spearman's Rho correlation test showed a difference in correlation coefficients for the different genders. While the male population showed a slightly positive and significant correlation between relative phase angle and cadence change (r = .124, p < .01, N = 363), the female population demonstrated a much higher correlation coefficient at a higher level of significance (r = .449, p < .001). These results indicate that females might indeed be more susceptible towards the influence of relative phase angles on cadence.

#### Gender differences: Motivation

With respect to gender, our interest was to find out if there are differences in entrainment capacity and effects on cadence. For completeness sake, we also report that no significant differences were observed for the enjoyment ratings (PACES) between men and women: not in general, and not when the data were inspected per strategy.

## Conclusions

Music-to-movement alignment strategies make it possible for everyone to keep running in synchrony with a musical stimulus, without consciously manipulating your own behavior to align with the music. Our study shows that strategies implementing a continuous relative phase alignment achieve the highest level of synchronization (as intended) and are therefore perfectly capable of spontaneously manipulating running behavior. A change in cadence is in fact induced by deviating the relative phase angle continuously from a preferred phase angle that is typically slightly negative: a more negative phase angle (step before beat) causes a slow-down in cadence and a positive phase angle (step after beat) leads to an increase in cadence.

Not only was cadence affected by phase angles that diverged from the runners’ preferred phase angles, also enjoyment levels were affected. Although differences in enjoyment were not very obvious, small, slightly significant increases in enjoyment were reported for the strategies that impacted cadence. Spontaneously guiding runners a little away from their preferred running behavior could add just enough challenge to their exercise, resulting in a more rewarding experience [39, 40].

The general preference for the footfalls to occur prior to the beats suggests that future music alignment strategies could be improved by considering the negative mean asynchrony (NMA) rather than 0° as a reference point. A question for future research might be whether the size of the NMA is task- and/or user dependent. For example, is human anticipation of a beat different when running compared to tapping? Are there differences between people and why? With respect to the latter, our results indicate that gender plays a role when using forced phase algorithms: effects of phase coherence and change in cadence were more pronounced for females compared to their male counterparts.

### Practical applications

Music-to-movement alignment strategies enable us to continuously and closely follow a person's behavioral response to music. This is of great value for sports and rehabilitation programs where music-based biofeedback is employed to improve individual performance [43]. In the future, the aim is to further improve our alignment strategies and introduce musical beats either slightly before or after the predicted footfalls. Such strategies could open up possibilities to spontaneously (and imperceptibly) optimize cadence and step size [44].
